# Reduction of *Alternaria* Toxins via the Extrusion Processing of Whole-Grain Red Sorghum Flour

**DOI:** 10.3390/foods13020255

**Published:** 2024-01-13

**Authors:** Elizabet Janić Hajnal, Janja Babič, Lato Pezo, Vojislav Banjac, Bojana Filipčev, Jelena Miljanić, Jovana Kos, Breda Jakovac-Strajn

**Affiliations:** 1 Institute of Food Technology, University of Novi Sad, Bulevar Cara Lazara 1, 21000 Novi Sad, Serbiabojana.filipcev@fins.uns.ac.rs (B.F.); jelena.krulj@fins.uns.ac.rs (J.M.); jovana.kos@fins.uns.ac.rs (J.K.); 2 Veterinary Faculty, University of Ljubljana, Gerbičeva 60, 1000 Ljubljana, Slovenia; janja.babic@vf.uni-lj.si (J.B.); breda.jakovacstrajn@vf.uni-lj.si (B.J.-S.); 3 Institute of General and Physical Chemistry, University of Belgrade, Studentski Trg 12–16, 11000 Belgrade, Serbia; latopezo@yahoo.co.uk

**Keywords:** co-rotating twin-screw extruder, red sorghum flour, alternariol, alternariol monomethyl ether, tenuazonic acid, tentoxin, UPLC-MS/MS

## Abstract

This study delved into the impact of two extrusion processing parameters—screw speed (*SS* at 400, 600, 800 RPM) and material moisture content in the extruder barrel (*M* at 12, 15, 18%) at constant feed rate (50 kg/h)—on reducing the content of alternariol (AOH), alternariol monomethyl ether (AME), tenuazonic acid (TeA), and tentoxin (TEN) in whole-grain red sorghum flour. Ultra-performance liquid chromatography combined with a triple-quadrupole mass spectrometer (UPLC-MS/MS) was employed for the determination of *Alternaria* toxin levels. The extruder die temperature fluctuated between 136 and 177 °C, with die pressures ranging from 0.16 to 6.23 MPa. The specific mechanical energy spanned from 83.5 to 152.3 kWh/t, the torque varied between 88 and 162.8 Nm, and the average material retention time in the barrel ranged from 5.6 to 13 s. The optimal parameters for reducing the concentration of all *Alternaria* toxins with a satisfactory quality of the sorghum snacks were: *SS* = 400 RPM, *M* = 12%, with a reduction of 61.4, 76.4, 12.1, and 50.8% for AOH, AME, TeA, and TEN, respectively.

## 1. Introduction

Sorghum (*Sorghum bicolor* L. Moench) is a grain-producing plant cultivated worldwide due to its high adaptability to various climates and its capacity to thrive in dry or arid environments. This crop demonstrates high photosynthetic efficiency and robustly withstands both drought and heat stresses, enabling its widespread cultivation across various geographical regions [1]. Sorghum is rich in micronutrients (minerals and vitamins) and macronutrients (carbohydrates, proteins, and lipids), as well as in phenolic compounds (flavonoids, phenolic acids, and tannins) which act as antioxidants [2]. Furthermore, sorghum belongs to the gluten-free cereals and its products demonstrate no toxicity for celiac patients in both in vitro and in vivo challenges. Hence, sorghum stands out as a secure dietary option for individuals diagnosed with celiac disease [3]. Sorghum is utilized for both human and animal consumption [1] due to its health benefits such as the inhibition of cancer cell growth, and reduction of obesity, heart disease, and diabetes [4,5]. It is versatile and can be utilized in baking, extrusion, and various cereal-based items like bread, cookies, expanded snacks, pasta, and breakfast cereals [2,6]. 

Despite being a significant source of nutrition for many communities, concerns have arisen in recent years regarding the presence of mycotoxins, harmful compounds produced by fungi. Sorghum is resistant to pests and microbes but susceptible to infection by different fungal species. It is prone to contamination by diverse mycotoxins, encompassing aflatoxins, *Fusarium* toxins (type A and B trichothecenes, fumonisins, zearalenone), and *Alternaria* toxins (such as alternariol (AOH), alternariol monomethyl ether (AME), tentoxin (TEN), and tenuazonic acid (TeA)), which are generated by *Aspergillus flavus*, *Fusarium* spp., and *Alternaria* spp., respectively [1]. Among these mycotoxins, *Alternaria* toxins have become the focal point of the presented research, as their concentrations in sorghum may pose risks to the health of both humans and animals. *Alternaria* toxins are known for their toxicity and have been associated with various adverse health effects, including carcinogenicity and immunosuppression [7]. 

The development of effective processes to reduce mycotoxin levels, especially those from the *Alternaria* toxin group, is therefore crucial for ensuring the safety and high quality of food products. Physical, chemical, and biological methods have been applied to prevent mycotoxin production or reduce mycotoxin levels [8]. Extrusion is an additional approach that could be used to reduce mycotoxin levels in sorghum and other cereals. It has been shown to be effective in reducing some mycotoxins in wheat and maize [8,9,10,11]. The first exploration into the potential reduction of *Alternaria* toxins in wheat through extrusion was published in 2016 [12]. Additionally, the same research group delved into the impact of the extrusion process on both *Fusarium* and *Alternaria* toxins in triticale flour, revealing their findings in a subsequent report in 2022 [13].

Extrusion is a high-temperature and high-pressure mechanical treatment of materials, characterized by its brief duration. This process is versatile, capable of processing various cereal products and animal feeds. Understanding the parameters affecting the efficacy of extrusion in mycotoxin removal is essential for the development of safe and high-quality sorghum or other cereal-based food products. This process requires the consideration of various extrusion parameters that influence toxin reduction while preserving the quality and nutritional value of the final product, such as temperature, raw material moisture, screw speed, and pressure [9,10,11]. The optimal temperature for reducing mycotoxins may vary depending on the type of mycotoxin and the raw material being processed. The pressure applied during extrusion is another crucial parameter affecting toxin reduction. High pressure can contribute to the mechanical destruction of the structure of mycotoxins, reducing their biological activity, but excessively high pressure may negatively impact the quality of the final product, such as by destroying nutrients. In addition to temperature and pressure, optimizing the duration of exposure is important for achieving effective toxin reduction while maintaining desired product characteristics [9,10,11].

Contemporary mathematical techniques like Response Surface Methodology (RSM), as highlighted by Grasso [14], offer a means to fine-tune the extrusion process. They enable the regulation of extrudate quality while assessing how extrusion variables affect mycotoxin reduction. Our study aims at optimizing the extrusion of whole-grain red sorghum flour. The main objective was to explore how altering extrusion parameters—specifically, the screw speed and material moisture content in the extruder barrel—affects both the mitigation of *Alternaria* toxins and the quality of the resulting extruded products.

## 2. Materials and Methods

### 2.1. Material Grinding and Mixing

Approximately 150 kg of red sorghum grain (*Sorghum bicolor* (L.) Moench) was commercially procured and finely ground using a hammer mill (ABC Inženjering, Pančevo, Srbija) equipped with a sieve of 1 mm in diameter. Before taking samples for analysis and extrusion, to achieve an appropriate level of homogeneity milled sorghum was blended in a Muyang SLHSJ0.2A twin-shaft mixer (Muyang, Yangzhou, China) for 90 s. In order to ensure the uniformity of sorghum flour mixing, the Microtracer^®^ method was employed, utilizing external tracers for homogeneity testing [15]. Additionally, eight subsamples were obtained to analyze the levels of *Alternaria* toxins (AOH, AME, TeA, and TEN) present in the whole-grain red sorghum flour.

### 2.2. Extrusion Conditions

A co-rotating twin-screw extruder (Bühler BTSK-30, Bühler, Uzwil, Switzerland), boasting a total barrel length of 880 mm with 7 sections and a length-to-diameter ratio of 28:1, was employed for sorghum extrusion. This extruder was equipped with two tempering tools to regulate water temperature for heating/cooling the barrel sections. The first tempering tool maintained a temperature of 80 °C for sections 2, 3, and 4, while the other controlled sections 6 and 7 at 120 °C. A die plate with a single 4 mm diameter opening and a cone inlet (yielding a total die area of 12.56 mm^2^) was utilized. Additionally, a specially engineered screw configuration tailored for directly expanded products was utilized in this process [16]. The dry material’s feeding rate was established at 50 kg/h, while adjustments to the screw speed and material moisture in the extruder barrel were made throughout the extrusion process according to a 3 × 2 full factorial design (Table 1). The water was added directly into section 2 of the extruder barrel by cavity pump, ensuring the desired moisture content of the material in it. The temperature and the pressure at the material outlet were measured by sensors that were mounted at the die head. All extrusion parameters, such as die temperature, die pressure, motor load (torque), and specific mechanical energy, were directly obtained from the extruder’s control screen. To achieve the desired product length, the outlet cutter of the extruder die was equipped with six knives rotating at a speed of 700 RPM. Following the extrusion process, the products were allowed to cool in ambient air and subsequently packed in nylon bags for further analysis.

### 2.3. Chemicals and Reagents

The individual standards for AOH, AME, TeA, and TEN (each with a purity ranging between 98.8 ± 1.5% and 99.5 ± 0.5%), sourced from Romer Labs in Tulln, Austria, were employed. Stock standard solutions and working standard solutions were both prepared in acetonitrile and stored in amber glass vials at −20 °C. Known concentrations of working standard solutions were prepared by appropriately diluting the stock standard solution. Acetonitrile, methanol (from Honeywell, Seelze, Germany), acetic acid (from Sigma-Aldrich, Steinheim, Germany), and ammonium acetate (from Merck, Darmstadt, Germany) were of p.a. or LC–MS purity. A Milli-Q system by Millipore in Bedford, MA, USA, was utilized to prepare deionized water.

### 2.4. Moisture Content

The moisture content in both the sorghum flour and extruded products was assessed using the established ISO 712/2009 standard method [17].

### 2.5. Sample Preparation for LC-MS/MS Analysis

The sample preparation involved a straightforward single-step extraction method, extensively detailed by Topi et al. [18] and Babič et al. [19].

### 2.6. LC-MS/MS Analysis

In order to quantify the tested *Alternaria* toxins (AOH, AME, TeA, and TEN), ultra-performance liquid chromatography coupled with a triple-quadrupole mass spectrometer (UPLC-MS/MS; Waters, Milford, MA, USA) was employed. This system featured an electrospray ionization (ESI) interface and utilized MassLynx software (version 4.2) for data acquisition and analysis (Waters, Milford, MA, USA), as extensively outlined by Topi et al. [18] and Babič et al. [19].

### 2.7. Expansion Ratio

To ascertain the expansion ratio (*ER*), the cross-sectional diameter of the extrudates was gauged using a sliding caliper equipped with a Vernier scale. This ratio was computed by dividing the cross-sectional diameter of the extrudate by the diameter of the die opening [20,21]. Ten random samples from each trial (extrusion condition) were used to derive the ER values.

### 2.8. Bulk Density of Extrudates

Measurement of the bulk density of extrudates was carried out in triplicate using a bulk density tester (Tonindustrie, West und Goslar, Germany).

### 2.9. Texture Analysis

The textural attributes of the extrudates were assessed using a Texture Analyser (model TA.XTPlus, Stable Micro Systems Ltd., Godalming, Surrey, UK) outfitted with a 50 kg load cell. Samples, arranged on a single-layer bed, underwent compression using an Ottawa cell with a 17-bladed extrusion plate. The test conditions included a probe distance of 57 mm, a pre-test speed of 5 mm/s, and a post-test speed of 10 mm/s. Each measurement was performed with 20 replicates. The obtained multi-peak force–time curve was used to extract several parameters as indicators of sample crispness and stiffness. The compression curve of crispy samples is characterized by a lot of fluctuations due to multiple fracture events. Indicators for crispness included two parameters: linear distance and the count of fractures. Linear distance on the compression curve represents the length of an imaginary line connecting all points within the specified region. The count of fractures corresponds to the number of force peaks recorded during sample compression. Greater linear distance and a higher number of force peaks both signify greater sample crispness. Sample stiffness was calculated as an average gradient of all positive peaks (trough-to-peak) on all peak and trough pairs in the selected region of the curve. The gradient of each pair was summed and divided by the number of trough-to-peak slopes in the selected region. Higher values of average gradient indicate higher stiffness or a less deformable product.

### 2.10. Water Absorption Index (WAI) and Water Solubility Index (WSI)

The water absorption index (*WAI*) and water solubility index (*WSI*) were assessed following the procedure outlined by Anderson et al. [22], with minor adjustments, as detailed by Janić Hajnal et al. [13]. The results for *WAI* and *WSI* were presented as the average values obtained across 4 extruded products from each trial. The *WAI* and *WSI* of whole-grain red sorghum flour were 2.54 ± 0.01 (g/g) and 3.97 ± 0.22 (g/100 g), respectively.

### 2.11. Statistical Analysis

#### 2.11.1. Principal Component Analysis

Principal Component Analysis (PCA) was employed to elucidate and identify patterns within the collected data. Additionally, an Analysis of Variance (ANOVA) was conducted to specifically assess the influence of the factor variables on the responses. These ANOVA computations were executed using the experimental data via TIBCO Statistica^®^ 14.0.0.15 software [23].

#### 2.11.2. Response Surface Methodology

The extrusion process of whole-grain sorghum flour was influenced by two factor variables—SS (400, 600, 800 RPM) and M (12, 15, 18%)—according to the experimental plan detailed in Table 1. These factor ranges were established based on preliminary trials. Utilizing a design featuring three levels for each of the two factors, the gathered experimental data were analyzed to assess their effects. With a limited sample size of 9, this proved sufficient for evaluating second-order polynomial (SOP) coefficients [24].

#### 2.11.3. Standard Score

Standard scores were assessed across various mycotoxin reduction trials conducted using the extrusion process. The ranking method relied on the ratio between the raw data and the extreme values for each response [13].

## 3. Results

### 3.1. Evaluation of the LC-MS/MS Method

To assess the accuracy of the analytical method, recovery studies were conducted. The recovery (*R*) for each *Alternaria* toxin in both matrices (whole-grain red sorghum flour and extruded product samples) was determined by dividing the slope of the spiked sample-prepared curve (a*_SP_*) by the slope of the matrix-matched calibration (a*_MMC_*) as follows:

*R* (%) = 100 × a*_SP_*/a*_MMC_* (1)

The spiked sample-prepared and matrix-matched calibration curves were prepared in the range of concentration presented in Table 2, at four concentration levels for each *Alternaria* toxin (Table 3) following the methodology detailed in our prior investigations [12,25]. 

It can be seen (in Table 2) that the *R* for all target mycotoxins was above 70% in both matrices, satisfying the criterion that *R* should be between 70 and 120% [26].

To assess the method’s precision with whole-grain sorghum flour and extruded product samples, we evaluated their repeatability and within-laboratory reproducibility. These results, expressed as relative a standard deviation (RSDr and RSD_wR_), were in the range of 2.6–11.8% and 3.5–15.7%, respectively, thus fulfilling the criteria of RSDr ≤ 20% and RSD_wR_ ≤ 20 [26], indicating the good precision of the method used (Table 3).

The limit of detection (LOD) for individual analytes was established at a signal-to-noise ratio of 3:1, while a value 3.3 times higher than the LOD was set as the limit of quantification (LOQ) [26]. The LODs and LOQs for both matrices (whole-grain red sorghum flour and extruded product samples) were as follows: 3.8 µg/kg and 12.5 µg/kg for AOH, and 1.9 µg/kg and 6.25 µg/kg for AME, TEN, and TeA, respectively.

### 3.2. Determination of Alternaria Toxins’ Levels

The quantification of the examined *Alternaria* toxins involved an external matrix-matched calibration method to counteract any matrix effects. Separate calibrations were prepared for both the whole-grain red sorghum flour and extruded product samples. The resulting data were adjusted for recovery (*R*) and expressed based on dry matter content. In the naturally contaminated whole-grain red sorghum flour, the initial water content, calculated on a dry weight basis, stood at 10.4 g/100 g. The initial concentrations (averages of eight measurements) of the examined *Alternaria* toxins were as follows: 111.4 ± 4.72 µg/kg for AOH, 30.2 ± 5.70 µg/kg for AME, 412.5 ± 11.7 µg/kg for TeA, and 30.6 ± 3.69 µg/kg for TEN. All extruded product samples underwent duplicate analysis, with their water content on a dry weight basis ranging from 6.2 to 12.2 g/100 g across nine samples. In these extrudates, the final concentrations of the examined *Alternaria* toxins varied between 42.2 and 43.9 µg/kg for AOH, 6.02 and 8.7 µg/kg for AME, 362 and 400 µg/kg for TeA, and 13.3 and 17.4 µg/kg for TEN.

### 3.3. Reduction of Alternaria Toxins due to Extrusion Processing

The extrusion process involved varying the screw speed and material moisture within the extruder barrel, following a 3 × 2 full factorial design (as outlined in Table 1), while maintaining a constant feed rate of 50 kg/h. Throughout extrusion, the reduction rate of the analyzed mycotoxins was calculated as a percentage reduction relative to their initial concentrations in the whole-grain red sorghum flour. This reduction rate served as a key parameter in all subsequent statistical analyses. The determined responses (*T*, *P*, *SME, Torque*, *t*, AOH, AME, TeA, and TEN reduction rates, *ER, BD*, H, *CLD, NoCP*, *CAG*, *WAI*, and *WSI*), as effects of the extrusion process variables (*M* and *SS*), are shown in Table 4. 

The co-rotating twin-screw extruder displayed varying responses within the following ranges: temperature (*T*) ranged from 136 to 177 °C, pressure (*P*) from 0.16 to 6.23 MPa, specific mechanical energy (*SME*) from 83.5 to 152 kWh/t, torque from 88.0 to 163 Nm, and the mean retention time in the barrel (*t*) ranged from 5.6 to 13 s. During the extrusion processing, the concentrations of all examined *Alternaria* toxins were reduced (Table 4). The reduction of AOH ranged from 60.6 to 62.1%, while for AME, TeA, and TEN it ranged from 71.1 to 80.0%, from 3.14 to 12.1%, and from 43.1 to 56.7%, respectively. As can be seen from Table 4, the maximum reduction of the examined *Alternaria* toxins was achieved at different process parameters during the extrusion of sorghum flour. The maximum reduction rate for AOH of 62.1% was obtained at *M* = 15% and *SS* = 400 RPM (sample 6), while for AME a reduction rate of 80.0% at *M* = 15% and *SS* = 800 RPM (sample 4) was recorded. The most significant reduction rate (12.1%) for TeA was achieved at the lowest moisture level in the extruder barrel (12%) and the lowest screw speed (400 RPM) (sample 7). On the other hand, the highest reduction rate for TEN, reaching 56.7% (sample 2), was obtained with the highest moisture level in the extruder barrel (18%) and a medium screw speed (600 RPM). Further, the range of values of the physico-chemical quality indicators of sorghum snacks in response to the applied process parameters were: an *ER* from 1.88 to 3.76, *BD* from 0.048 to 0.318 g/mL, *H* from 9.53 to 23.9 kg, *CLD* from 51.6 to 61.8 kg.s, *NoCP* from 4.48 to 56.8, *CAG* from 10.9 to 17.9 kg/s, *WAI* from 3.82 to 5.81 g/g, and *WSI* from 8.73 to 31.1 g/100 g.

According to the main objective of this study, the results of the applied contemporary mathematical techniques are presented below. 

#### 3.3.1. Principal Component Analysis

**Figure 1:**
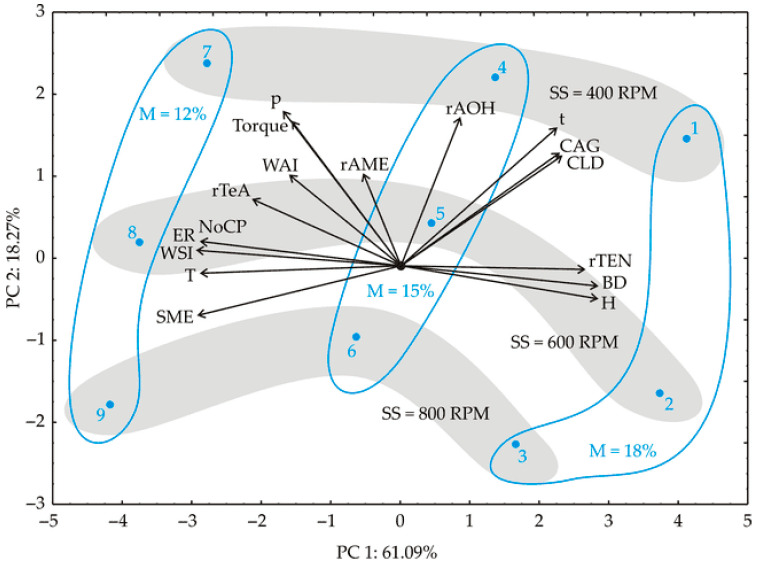
PCA ordination of variables based on component correlations, presented in the first and the second factor plane. *M*—moisture of the material in the extruder barrel (%); *SS*—screw speed (RPM); *T*—die temperature (°C); *P*—pressure at the die (MPa); *SME*—specific mechanical energy (Wh/kg); *Torque* (Nm); *t*—mean retention time in the barrel (s), *r*AOH—reduction of alternariol (AOH) (%); *r*AME—reduction of alternariol monomethyl ether (AME) (%); *r*TeA—reduction of tenuazonic acid (TeA) (%); *r*TEN—reduction of tentoxin (TEN) (%), *BD*—bulk density (g/mL); *H*—snack hardness (kg); *CLD*—crispness by linear distance (kg.s); *NoCP*—crispness by number of fractures; *CAG*—stiffness by average gradient (kg/s); *WAI*—water absorption index (g/g); *WSI*—water solubility index (g/100 g).

Initially, when the collected experimental dataset underwent PCA analysis, distinct groupings of samples emerged based on the factor variables. As an exploratory tool, this method facilitated the description and differentiation of response variables (seen in Figure 1). The results of the PCA analysis interpreted the initial two principal components, explaining 79.4% of the total variance, which is considered sufficient for explaining the data. Notably, certain factors, namely *r*TEN, *BD*, and *H*, held greater significance in the assessment of the first principal component (PC1), positively contributing 7.4%, 8.5%, and 8.5%, respectively, based on correlations, while the variables *T*, *SME*, *NoCP*, *ER*, and WSI influenced the PC1 calculation negatively, providing 8.8%, 8.9%, 8.8%, 9.4%, and 9.2%, of the total variance, respectively. On the other hand, factors such as *P*, *Torque*, *t*, *r*AOH, *CLD*, and *CAG* played more crucial roles in the computation of the second principal component (PC2), positively contributing 17.0%, 14.8%, 14.4%, 16.3%, 9.4%, and 9.5%, respectively.

The PCA plot in Figure 1 vividly displays the separate groupings of the samples. Those processed with a lower moisture content (7, 8, and 9) are distinctly situated on the left-hand side of the chart. These samples exhibit higher levels of *P*, *T*, *Torque*, *SME, WAI*, and *WSI,* and display a notable decrease in TeA content. Conversely, the samples produced with a higher moisture content (1, 2, and 3) showcase increased *r*TEN, *BD*, and H values. Additionally, samples processed with lower screw speed values exhibit elevated values for *r*AOH, t, *CAG*, and *CLD*.

#### 3.3.2. Response Surface Method

An ANOVA analysis was applied to the second-order polynomial (SOP) models to investigate the impacts of the input variables, as detailed in Table 5. The results highlighted that, in the SOP model for *T* computation, the linear terms of *SS* and *M* surfaced as the most impactful variables, demonstrating statistical significance at *p* < 0.001 and *p* < 0.05, respectively. For *P* evaluation, the SOP model highlighted the notable impact of the linear terms of *M* and *SS* (statistically significant at *p* < 0.01). In the *SME* calculation, the linear terms of *M* and *SS* held substantial influence (statistically significant at *p* < 0.001). Torque evaluation predominantly saw impact from the linear terms of *SS* and *M* in the SOP model (statistically significant at *p* < 0.01), while the quadratic term of SS and the interchange term of *M* × *SS* were also notable contributors (statistically significant at *p* < 0.01 and *p* < 0.05, respectively). The linear terms of moisture content and screw speed were statistically significant factors, at the *p* < 0.01 level, in the SOP model for *t* calculation. Further analysis revealed that the linear term of *M* significantly influenced the computation of *r*TEN (statistically significant at the *p* < 0.05 level). Meanwhile, *r*AOH, *r*AME, and *r*TeA were not significantly affected by the linear terms of *SS* and *M* (*p* > 0.05 level).

The linear terms of *M* and *SS* within the SOP model significantly influenced *ER* calculation at *p* < 0.001. Moreover, the linear term of *M* exhibited a statistically significant influence on *H,* at *p* < 0.01. For *BD*, the SOP model was notably affected by the linear terms of *M* and *SS*, along with the quadratic term of *SS* (statistically significant at *p* < 0.001). Additionally, the non-linear term *SS* × *M* proved statistically significant at *p* < 0.01. In the SOP model for *CLD* calculation, the linear terms of moisture content and screw speed, as well as the quadratic term of moisture content, emerged as statistically significant factors at *p* < 0.01. Similarly, for *NoCP* calculation, the linear term of *M* showed statistical significance at *p* < 0.01.

Regarding *CAG* calculation, both the linear terms of *M* and *SS* were statistically significant at *p* < 0.001, with the quadratic term of SS also proving significant at *p* < 0.05. For *WSI*, the linear terms of *M* and *SS* exhibited significance at *p* < 0.001 and *p* < 0.01, respectively, while the combined effect of *M* × *SS* reached statistical significance at *p* < 0.05. All SOP models passed lack-of-fit tests, ensuring their adequate representation of the data. Furthermore, the high *r*^2^ values confirmed a strong correlation between the model predictions and experimental results. In the optimization of extruder parameters using standard scores, the optimal score was computed by averaging the scores across all mycotoxin reduction variables. The maximal score function revealed the ideal factor variables and optimal levels for reducing mycotoxins. 

**Figure 2:**
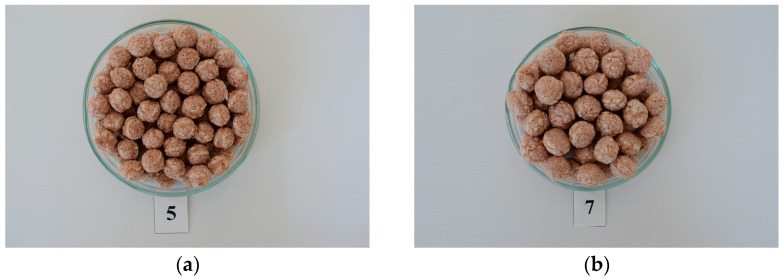
Sorghum snacks with the highest scores. (**a**) Sample 5 at S = 0.69; (**b**) Sample 7 at S = 0.68.

These results are illustrated in Table 4 (the column Score). The highest scores were attained in sample 5 (Figure 2a), with the optimized parameters set as follows: *SS* = 600 RPM and *M* = 15 g/100 g. Under these conditions, the extrusion process resulted in *T* = 165 °C, *P* = 1.78 MPa, *SME* = 117.3 Wh/kg, *Torque* = 99 Nm, and *t* = 9.4 s. Additionally, the physicochemical properties of the optimal sample were determined: *ER* = 2.84, *BD* = 0.12 g/mL, *H* = 16.35 kg, *CLD* = 60.66 kg·s, *NoCP* = 20.67, *CAG* = 15.27 kg/s, *WAI* = 4.55 g/g, and *WSI* = 20.45 g/100 g (refer to Table 4). In these optimal extrusion conditions, the reduction rates of the examined mycotoxins were as follows: 62.0% for AOH, 76.1% for AME, 8.76% for TeA, and 51.4% for TEN.

Sample 7 (Figure 2b) closely followed the optimal score, achieving a score of 0.68 (Table 4). Sample 7 was produced with extruder parameters set at *M* = 12% and *SS* = 400 RPM, resulting in *T* = 168 °C, *P* = 6.23 MPa, *SME* = 132.2 Wh/kg, *Torque* = 162.8 Nm, and *t* = 9.5 s. The physicochemical properties of this optimal sample were determined: *ER* = 3.58, *BD* = 0.07 g/mL, *H* = 11.85 kg, *CLD* = 56.29 kg·s, *NoCP* = 41.80, *CAG* = 14.44 kg/s, *WAI* = 5.40 g/g, and *WSI* = 30.08 g/100 g (refer to Table 4). Regarding the *Alternaria* toxins’ reduction achieved with these extrusion conditions, sample 7 demonstrated reductions of 61.4% for AOH, 76.4% for AME, 12.1% for TeA, and 50.8% for TEN.

As can be seen in Figure 2a,b and Table 4, sample 7 had better quality attributes of its extrudate (*ER, BD, H, CLD, NoCP, CAG, WAI, WSI*), as well as a slightly lower reduction of AOH, AME, and TEN contents and a greater degree of reduction in its TeA content compared to sample 5 (see Section 3.3.2).

For the above-mentioned reasons, the optimal conditions of the extrusion process were chosen to correspond to sample 7, since under the given conditions, a reduction of *Alternaria* toxins and a satisfactory quality of the sorghum snack were simultaneously achieved.

## 4. Discussion

To the best of the authors’ knowledge, no previously published data exist regarding the behavior of co-occurring *Alternaria* toxins in sorghum during the extrusion process. Studies regarding the fate of *Alternaria* toxins during the extrusion of cereals are rare and mainly refer to our previous investigations [12,13]. The AOH reduction rates depended on the applied process parameters of the pilot scale co-rotating twin-screw extruder and ranged from 60.6 to 62.1%, while our earlier investigation concerning the extrusion of whole-grain wheat flour using a pilot-scale single-screw extruder [12] had a reduction rate that was higher by more than 10% (72.7 to 87.9%). Concerning the fate of AME during the extrusion process, its reduction rate in this study ranged from 71.1 to 80.0%, while in our previous studies [12,13] its reduction rate was quite similar. Namely, the reduction rate of AME in whole-grain triticale flour extruded using a twin-screw extruder ranged between 53.2% and 91.8%, contingent upon the extrusion parameters [13]. Conversely, in the extrusion of whole-grain wheat flour via a single-screw extruder, the AME reduction rate varied from 62.8% to 94.5% [12]. In this current study, the reduction rate of TeA was notably lower, ranging from 3.14% to 12.1%. Interestingly, in the SOP model, the linear terms of *SS* and *M* did not significantly affect TeA reduction (*p* > 0.05 level). In contrast, during the extrusion of whole-grain wheat flour using a pilot-scale single-screw extruder, TeA reduction ranged from 40.3% to 62.5%. In this case, the simultaneous influence of moisture content and screw speed emerged as significantly crucial factors affecting TeA reduction at a significance level of *p* < 0.05 [12]. Further, by applying those process parameters in the present study, the reduction rate of TEN ranged from 43.1 to 56.7%, and its reduction was significantly influenced by the linear term of *M* (statistically significant at the *p* < 0.05 level) in the SOP model. Contrary to this finding, in the extrusion of whole-grain triticale flour [13] TEN’s reduction rate ranged between 1.7 and 21.2%, and was mostly influenced by the linear terms of *SS* and *MC*, and also by the quadratic term of *SS* and the non-linear term of *SS* × feed rate (*FR*) in the SOP model (*p* < 0.01 level). 

Based on the published data, it can be concluded that the level of *Alternaria* toxin reduction during extrusion depends on the interaction of numerous factors, as is the case with the other mycotoxins examined so far, which include the type of extruder, the extrusion conditions (screw speed, feed rate, extruder temperature, die temperature, pressure, and residence time in the extruder), the type and particle size of the raw materials or extrusion mixture, the moisture content of the raw materials or extrusion mixture in the extruder barrel, the chemical structure of the mycotoxins, and their initial content in the raw material [9,10].

Regarding the quality indicators for sorghum snacks, the expansion ratio (ER) stands out as the most critical attribute for assessing extruded snacks’ quality. The expansion process, occurring within a second at the die exit, encompasses several stages: bubble formation, growth, merging, shrinkage, and finally, solidification, as the starchy matrix turns glassy. During extrusion, post-mixing, and hydration, the starch undergoes melting due to both external heat and the viscous forces emerging from the die. Simultaneously, water evaporates, leading to instantaneous expansion, during which bubble growth halts, stopping expansion before the material solidifies. The cellular structure solidifies at a preset temperature. Under specific conditions—such as a setting temperature surpassing 100 °C and a low moisture content within the raw material in the extruder barrel—vapor bubbles may expand to a temperature beyond 100 °C, and the structure solidifies before bubble collapse. This phenomenon results in heightened expansion [27]. In this study, the setting temperature of sections 6 and 7 of the extruder barrel was set at 120 °C. An elevated expansion ratio (*ER*) was noted when the material within the extruder barrel had the lowest moisture content, coupled with a high screw speed and die temperature. (Table 4). These findings are in agreement with those reported by Llopart et al. [28] and Kaur et al. [29,30]. Further, the *ER* of the extrudates ranged between 1.88 and 3.76 (Table 4), and was significantly influenced by the linear term of the moisture of the material in the extruder barrel and screw speed at the *p* < 0.001 level in the SOP model. 

The *H* was mainly influenced by the moisture of the material in the extruder barrel at *p* < 0.01. Namely, with a decrease in the moisture of the material in the extruder barrel, a decrease in the *H* of sorghum snacks was observed. It is well known that water acts as a plasticizer to the starch-based material, reducing its viscosity and the mechanical energy dissipation in the extruder. For the reason mentioned, the product becomes dense and bubble growth is compressed [31,32]. It can be observed from Table 4 that the hardness was lower with the decreasing moisture content of the material in the extruder barrel and the increasing screw speed, which were accompanied by an increase in the die temperature of the extruder. These findings are in agreement with previous studies [28,29,30,33].

Bulk density serves as a gauge for the expansion that takes place during extrusion and holds significant importance as a quality indicator for extruded products [34]. In the case of sorghum snacks, the bulk density (*BD*) was notably influenced by the linear terms of moisture content (*M*) and screw speed (*SS*), alongside the quadratic term of SS within the SOP model (significantly significant at *p* < 0.001). Additionally, the non-linear term *SS* × *M* showed statistical significance at *p* < 0.01. Similar correlations between *BD* and the extrusion process parameters were noted in a study on brown rice grits by Pardhi et al. [33]. The BD of sorghum extrudates ranged from 0.048 to 0.318 g/mL (Table 4). With a decrease in the moisture of the material in the extruder barrel and by increasing the screw speed during a constant feed rate (50 kg/h), the *BD* of the sorghum extrudates decreases, which indicates a greater expansion during the extrusion process. Several studies have demonstrated a high positive correlation between bulk density and hardness [29,30,35]. 

The textural attributes of the sorghum extrudates underwent notable alterations due to the extrusion process parameters. Both the crispness and stiffness of the extrudates experienced substantial changes due to the linear effects of the independent variables: moisture and screw speed. The crispness parameter indicated by the linear distance was also significantly affected by the quadratic effect of moisture. Crispness, indicated by the number of fractures on the compression curve, was highly impacted by the linear effect of moisture at the *p* < 0.001 significance level. Besides the linear effect of moisture, the extrudate stiffness was significantly affected by the linear and quadratic effects of screw speed. All detected relationships were positive, meaning that increased moisture levels and screw speeds increased the stiffness and crispness of the extrudates. Stiffness is a parameter closely related to hardness. Stiff materials are hard and not prone to easy deformation. In other studies, a trend of increased hardness with higher moisture levels was also observed, which might be due to lower expansion. In our study, screw speed significantly positively affected stiffness. This is in contrast to the reported lower hardness found with increased SS in barley and corn extrudates [31,32] and chick-pea flour extrudates [36]. In the sorghum extrudates in this study, increasing the moisture level and screw speed led to the formation of a more complex inner structure consisting of firm walls of pores that gave highly jagged compression curves as a result of many fracture events during the compression of pore walls. The harder and more compact structure of extrudates was related to an increased crispness in the microstructural study of Dar et al. [37] regarding extrudates based on rice flour, pulse powder, and carrot pomace. Wojtowicz et al. [38] observed a strong positive correlation between the hardness and fracturability of buckwheat-enriched extrudates, but crispness did not follow consistent trends.

The water absorption index (WAI) gauges the quantity of water absorbed by starch, serving as an indicator of gelatinization. It depends on the presence of hydrophilic groups that facilitate the binding of water molecules. The *WSI* measures the amount of soluble components released from the starch (amount of soluble polysaccharide) after extrusion, and the WSI is often used as an indicator of the degradation of molecular components, which is a measurement of the degree of conversion of starch during extrusion, due to gelatinization and dextrinization [20]. The *WAI* and the *WSI* of whole-grain red sorghum flour were 2.54 ± 0.01 g/g and 3.97 ± 0.22 g/100 g, while in extrudates they ranged between 3.82 and 5.81 g/g and 8.73 and 31.1 g/100 g, respectively. As Llopart et al. [28] reported, the *WAI* and *WSI* of the whole-grain red sorghum extrudate have a direct relationship to temperature and an inverse relationship to moisture. Based on previously published data for the *WAI* and *WSI* of extruded products using mathematical modeling, Oikonomou and Krokida [39] reported that the *WAI* increases sharply with feed moisture content for all of their extruded food products, except for wheat and oat products. In our investigation, using PCA, samples generated with a lower moisture content (samples 7, 8, and 9) displayed elevated levels of *WAI* and *WSI* (refer to Figure 1). Interestingly, the *WAI* did not show significant influence from the linear terms of *SS* and *M* (*p* > 0.05 level). The *WSI* was influenced by the linear terms of *M* and *SS* (*p* < 0.001 and *p* < 0.01, respectively), while the impact of the product term *M* × *SS* was statistically significant at the *p* < 0.05 level in the SOP model. In our study, with an increase in the moisture content of the material in the extruder barrel and a decrease in the screw speed at all applied moisture contents of the material in the extruder barrel, a decrease in the WSI was observed (Table 4). Our findings are in agreement with the statement by Oikonomou and Krokida [39], that an increase in the moisture of the material in the extruder barrel leads to a slight decrease in the *WSI* for all food products, except beans. 

In this investigation, reducing the material’s moisture content within the extruder barrel and augmenting the screw speed, followed by increasing the die temperature, led to a decrease in the *H*, *BD*, *CAG*, and *CLD*, and to an increase in the *ER* and *WSI* of the extrudates. These findings are in agreement with the findings reported by Kaur et al. [29,30], who demonstrated a high negative correlation between *ER* and *BD*, as well as between *ER* and *H*, and a high positive correlation between *ER* and *WSI* during evaluation of the effect of extrusion processing on the techno-functional properties of sorghum–chickpea-based extruded snacks and sorghum–mung bean combination snacks.

## 5. Conclusions

Extrusion in this study is presented as a highly promising process for reducing *Alternaria* toxins in food products, allowing the food industry to provide safe, healthy, and high-quality products to consumers while minimizing the adverse effects of mycotoxins on human health. The best standard score (*Score* = 0.689) was obtained for sample 5, by employing extrusion process parameters of a moderate moisture content in the raw material within the extruder barrel (*M* = 15%) and a medium screw speed (*SS* = 600 RPM) at constant feed rate (*FR* = 50 kg/h), which provide the optimal reduction rates of the present *Alternaria* toxins in the final product, but the quality indicators of the sorghum snack were not completely satisfactory. Thus, the optimal *Score* = 0.681 was adopted, which was achieved for sample 7, by using the extrusion process parameters with the lowest moisture content of raw material in the extruder barrel (*M* = 12%) and the lowest screw speed (*SS* = 400 RPM), at a constant feed rate (*FR* = 50 kg/h), simultaneously ensuring a satisfactory reduction of the *Alternaria* toxins’ content (61.4% for AOH, 76.4% for AME, 12.1% for TeA, 50.8% for TEN) and a satisfactory quality (*ER* = 3.58, *BD* = 0.07 g/mL, *H* = 11.85 kg, *CLD* = 56.29 kg·s, *NoCP* = 41.80, *CAG* = 14.44 kg/s, *WAI* = 5.40 g/g, and *WSI* = 30.08 g/100 g) of final sorghum snack. The fact that the raw materials for extrusion (grains, legumes, oilseeds, etc.) are often simultaneously contaminated with several mycotoxins, while a complex interaction occurs among many parameters during the extrusion process, necessitates a thorough investigation into the extrusion process’s impact on distinct mycotoxins. This examination is essential for every combination of ingredient compositions and applied parameter settings. Furthermore, it is crucial to understand the fate and behavior of these toxins, especially during processing and treatment procedures. To prevent the potential risks associated with treated grains, an understanding of the chemical changes regarding the fate of toxins and the toxicity of their degradation products during food processing should be considered in future studies.

## Figures and Tables

**Table 1:** Independent extrusion parameters and their levels.

Experimental Factor	Factor’s Level		
	(Low)	(Center)	(High)
Screw speed (RPM)	400	600	800
Moisture content (%)	12	15	18

**Table 2:** Recovery data of the employed analytical method.

Analytes	Spiking Level (µg/kg) *	*R* (%) ** of Whole-Grain Red Sorghum Flour	*R* (%) ** of Extruded Product
AOH	12.5–100	90.1	102
AME	6.25–50	95.3	102
TeA	6.25–50	99.1	98.7
TEN	6.25–50	100	97.8

AOH—alternariol; AME—alternariol methyl ether; TeA—tenuazonic acid; TEN—tentoxin. * The concentration range of analytes for standard calibration curves, matrix-matched calibration curves, and the calibration curves of spiked samples is expressed in micrograms per kilogram (µg/kg). ** The recovery percentage (*R*) is calculated by dividing the slope of the spiked sample-prepared curve by the slope of the matrix-matched calibration curve.

**Table 3:** Precision of the analytical method used for determination of *Alternaria* toxins.

Analytes	Spiking Level (µg/kg)	Repeatability (*n* = 6) RSDr (%)	Within-Laboratory Reproducibility (*n* = 3 × 6) RSD_wR_ (%)	Repeatability (*n* = 6) RSDr (%)	Within-Laboratory Reproducibility (*n* = 3 × 6) RSD_wR_ (%)
		Whole-grain sorghum flour		Extruded product	
AOH	12.5	9.76	15.7	11.8	12.9
	25.0	9.67	12.4	9.31	9.96
	50.0	9.53	12.0	9.44	9.76
	100	8.05	9.16	8.3	8.5
AME	6.25	10.0	14.4	10.0	14.4
	12.5	9.45	13.1	9.45	13.1
	25.0	5.73	7.69	5.73	7.69
	50.0	5.11	6.82	5.11	6.82
TeA	6.25	9.74	12.6	10.8	13.4
	12.5	9.10	10.9	7.94	10.8
	25.0	7.43	9.80	7.04	9.20
	50.0	7.28	9.30	4.05	5.33
TEN	6.25	6.98	10.1	8.53	12.2
	12.5	3.55	4.00	6.16	10.9
	25.0	2.73	3.85	4.85	9.34
	50.0	2.62	3.50	3.91	8.33

AOH—alternariol; AME—alternariol methyl ether; TeA—tenuazonic acid; TEN—tentoxin; RSD—relative standard deviation.

**Table 4:** Technological parameters of extrusion, reduction of *Alternaria* toxins, and quality indicators of extrudates.

			Process Responses						Product Responses											
Sample	*M*	*SS*	*T*	*P*	*SME*	*Torque*	*t*	*rAOH*	*rAME*	*rTeA*	*rTEN*	*ER*	*BD*	*H*	*CLD*	*NoCP*	*CAG*	*WAI*	*WSI*	*Score*
1	18	400	136	2.65	83.5	114	13	61.4	75.0	5.45	55.4	1.88	0.318	23.9	61.8	4.48	17.9	5.71	8.73	0.536
2	18	600	144	1.25	99.6	88.0	10	60.6	71.3	4.55	56.7	2.02	0.278	21.6	60.3	5.70	17.2	3.82	11.8	0.302
3	18	800	153	0.16	112	99.0	8	61.1	74.9	3.14	55.7	2.15	0.222	18.5	53.6	6.35	12.9	4.44	15.7	0.423
6	15	400	159	4.03	104	134	12	62.1	77.5	3.56	52.5	2.68	0.145	15.4	61.7	18.4	16.8	4.72	20.0	0.613
5	15	600	165	1.78	117	99.0	9.4	62.0	76.1	8.76	51.4	2.84	0.115	16.3	60.7	20.7	15.3	4.55	22.4	0.689
4	15	800	166	1.28	130	114	8.5	60.9	80.0	6.89	54.7	2.97	0.090	17.6	55.3	19.6	12.1	4.69	26.1	0.619
7	12	400	168	6.23	132	163	9.5	61.4	76.4	12.1	50.8	3.58	0.065	11.8	56.3	41.8	14.4	5.40	30.1	0.681
8	12	600	176	4.81	140	117	7.7	60.7	78.2	7.91	45.7	3.63	0.055	9.53	52.8	56.8	13.6	5.81	31.1	0.405
9	12	800	177	3.04	152	123	5.6	60.6	71.1	8.75	43.1	3.76	0.048	12.1	51.6	50.7	10.9	5.42	30.5	0.156

*M*—moisture of the material in the extruder barrel (%); *SS*—screw speed (RPM); *T*—die temperature (°C); *P*—pressure at the die (MPa); *SME*—specific mechanical energy (Wh/kg); *Torque* (Nm); *t*—mean retention time in the barrel (s), *r*AOH—reduction of alternariol (AOH) (%); *r*AME—reduction of alternariol monomethyl ether (AME) (%); *r*TeA—reduction of tenuazonic acid (TeA) (%); *r*TEN—reduction of tentoxin (TEN) (%), *ER*—expansion ratio; *BD*—bulk density (g/mL); *H*—snack hardness (kg); *CLD*—crispness by linear distance (kg·s); *NoCP*—crispness by number of fractures; *CAG*—stiffness by average gradient (kg/s); *WAI*—water absorption index (g/g); *WSI*—water solubility index (g/100 g).

**Table 5:** ANOVA evaluation of the technological parameters and reduction of mycotoxins (sum of squares).

	*M*	*M2*	*SS*	*SS2*	*M × SS*	*Error*	*R2*
*df*	1	1	1	1	1	3	
*T*	1290.7 ***	37. 6	181.5 *	6.7	16.0	17.8	0.989
*P*	1673.3 **	87.1	1184.4 **	16.2	12.3	38.7	0.987
*SME*	2799.4 ***	13.9	917.6 ***	0.0	18.1	6.2	0.998
*Torque*	1706.9 **	4.3	932.5 **	1101.4 **	146.4 *	42.9	0.989
*t*	11.2 **	2.0	25.6 **	0.3	0.3	0.6	0.984
*rAOH*	0.0	1.0	0.9	0.0	0.1	0.6	0.773
*rAME*	3.3	22.7	1.3	0.8	6.6	35.3	0.495
*rTeA*	40.8	0.7	1.0	0.4	0.3	25.0	0.632
*rTEN*	133.1 *	5.1	4.5	1.1	15.8	15.3	0.913
*ER*	4.0 ***	0.0	0.9 ***	0.0	0.0	0.0	0.999
*BD*	0.1 ***	0.1 ***	0.1 ***	0.0	0.1 **	0.0	0.999
*H*	154.8 **	0.1	1.5	1.1	8.2	10.5	0.940
*CLD*	37.6 *	19.5 *	61.6 *	3.1	3.0	5.6	0.957
*NoCP*	2938.1 ***	130.6	24.1	34.3	12.6	46.6	0.985
*CAG*	13.6 **	0.1	29.4 ***	2.9 *	0.5	0.5	0.990
*WAI*	1.2	0.4	0.3	0.2	0.4	1.1	0.702
*WSI*	511.8 ***	4.5	30.6 **	0.0	10.8*	2.6	0.995

*** statistically significant at *p* < 0.001 level, ** statistically significant at *p* < 0.01 level, * statistically significant at *p* < 0.05 level. *M*—moisture of the material in the extruder barrel (%); *SS*—screw speed (RPM); *T*—die temperature (°C); *P*—pressure at the die (MPa); *SME*—specific mechanical energy (Wh/kg); *Torque* (Nm); *t*—mean retention time in the barrel (s), *r*AOH—reduction of alternariol (AOH) (%); *r*AME—reduction of alternariol monomethyl ether (AME) (%); *r*TeA—reduction of tenuazonic acid (TeA) (%); *r*TEN—reduction of tentoxin (TEN) (%); *ER*—expansion ratio; *BD*—bulk density (g/mL); *H*—snack hardness (kg); *CLD*—crispness by linear distance (kg·s); *NoCP*—crispness by number of fractures; *CAG*—stiffness by average gradient (kg/s); *WAI*—water absorption index (g/g); *WSI*—water solubility index (g/100 g).

## Data Availability

Data is contained within the article.
